# SGLT2 Inhibitors in Heart Failure with Reduced Ejection Fraction: A Retrospective Cohort Analysis of Sex-Specific Cardiovascular Outcomes

**DOI:** 10.2174/011573403X394902250911111958

**Published:** 2025-10-02

**Authors:** Ramzi Ibrahim, Rahmeh Al-Asmar, Hashim AlHammouri, Mahmoud Abdelnabi, Beani Forst, Hoang Nhat Pham, Patrick Sarkis, Steven J. Lester, Chadi Ayoub, Kwan Lee, Julie Rosenthal, Reza Arsanjani

**Affiliations:** 1 Department of Cardiovascular Medicine, Mayo Clinic, Phoenix, AZ 85054, United States of America;; 2 Faculty of Medicine, University of Jordan, Amman, Jordan;; 3 Department of Medicine, University of Arizona, Tucson, AZ 85721, United States of America;; 4 Department of Cardiovascular Medicine, Mayo Clinic, Rochester, MN 55905, United States of America

**Keywords:** Heart failure, disparities, guideline-directed medical therapy (GDMT), arrhythmias, propensity score matching (PSM), comorbidity burden

## Abstract

**Introduction:**

Sex-based differences in outcomes among patients with heart failure with reduced ejection fraction (HFrEF) treated with sodium-glucose cotransporter 2 inhibitors (SGLT2is) remain underexplored. This study aimed to evaluate sex-specific differences in cardiovascular outcomes in patients with HFrEF receiving SGLT2 inhibitors alongside guideline-directed medical therapy (GDMT).

**Methods:**

We conducted a retrospective cohort study using the TriNetX global research network. Adults with HFrEF treated with SGLT2is and GDMT were stratified by sex. Propensity score matching (PSM) was used to balance baseline demographics, comorbidities, medications, and laboratory data. Primary outcomes were all-cause mortality and acute heart failure (HF) events; secondary outcomes included hospitalizations, arrhythmias, renal outcomes, and advanced therapies.

**Results:**

After PSM, 17,408 male and 17,408 female patients were analyzed. Male patients had lower odds of acute HF events (aOR: 0.949; 95% CI: 0.909–0.991), all-cause hospitalizations (aOR: 0.933; 95% CI: 0.895–0.973), and renal failure (aOR: 0.915; 95% CI: 0.870–0.962). No significant differences were observed in all-cause mortality (aOR: 1.003; 95% CI: 0.926–1.087) or heart transplantation, although LVAD use was more frequent in males (aOR: 1.416; 95% CI: 1.053–1.905).

**Discussion:**

The findings highlighted potential sex-based disparities in outcomes for patients with HFrEF on SGLT2is. Differential prescribing patterns, comorbidity burden, or timing of therapy initiation may contribute to observed differences.

**Conclusion:**

Among HFrEF patients treated with SGLT2is, males experienced lower risks of HF events, hospitalizations, and renal failure compared to females, despite similar mortality. Further research is needed to understand and address sex-specific disparities in HFrEF management.

## 1 INTRODUCTION

Heart failure with reduced ejection fraction (HFrEF) poses a significant global health burden [1]. Despite advances in guideline-directed medical therapy (GDMT), including optimized pharmacological and device-based interventions, patients with HFrEF remain at high risk for adverse cardiovascular outcomes, such as recurrent hospitalizations, worsening heart failure, and mortality. Sodium-glucose cotransporter 2 inhibitors (SGLT2is), originally developed to manage glycemic control in type 2 diabetes mellitus (T2DM), have demonstrated remarkable cardiovascular benefits beyond glucose-lowering effects [2-7]. These benefits include reductions in major adverse cardiovascular events (MACE), heart failure-related hospitalizations, and mortality in patients with heart failure with either a preserved or reduced ejection fraction, irrespective of T2DM status [2-5, 8].

While the use of SGLT2is has increased in recent years, sex-specific variations in their efficacy and associated clinical outcomes in HFrEF remain underexplored [9-12]. Evidence suggests notable sex-specific differences in treatment response among patients with HFrEF, raising critical questions about how biological sex may influence outcomes [13]. Differences in GDMT initiation, dose-response, myocardial remodeling, hemodynamic responses, and hormonal interactions may modify the therapeutic effects of SGLT2is [14].

Although clinical trials investigating SGLT2is in populations with HFrEF are clinically diverse, large-scale cohort studies focusing on sex-specific differences in HFrEF-related outcomes for patients taking SGLT2is are lacking. Addressing this gap is critical to optimize treatment strategies and promote equitable care for patients with HFrEF. To address this, our study aimed to evaluate the sex-based impact of SGLT2is on cardiovascular outcomes in patients with HFrEF.

## 2 MATERIALS AND METHODS

This study adhered to the Strengthening the Reporting of Observational Studies in Epidemiology (STROBE) guidelines for observational cohort studies. Since de-identified and aggregated data were utilized from a publicly accessible federated research platform, the study did not require informed consent after review from the Mayo Clinic Institutional Review Board (protocol number: 24-013396). No direct human interaction or interventions were performed as this research study constituted a secondary analysis of existing data. In line with HIPAA privacy rule standards, all data de-identification protocols were rigorously followed.

### 2.1 Data Source

Our retrospective cohort analysis leveraged the TriNetX Analytics Research Network, a global federated database aggregating electronic medical records from over 130 million patients across 103 healthcare organizations. This repository permits analysis only of de-identified, aggregate-level data, in compliance with Section 164.514(a) of the Health Insurance Portability and Accountability Act (HIPAA) Privacy Rule. Our study was completed in accordance with STROBE guidelines.

### 2.2 Study Design and Population

This study was conducted in accordance with the Sex and Gender Equity in Research (SAGER) guidelines. We analyzed adult patients (aged ≥18 years) with HFrEF stratified by sex (male *versus* female) who were on SGLT2is in combination with GDMT [angiotensin-converting enzyme inhibitors/angiotensin receptor blockers/sacubitril and valsartan, mineralocorticoid receptor antagonists (spironolactone or eplerenone), and beta-blockers (metoprolol or carvedilol)]. We utilized data recorded from January 1, 2012, through December 31, 2022. Propensity score matching (PSM) was applied to adjust for baseline differences in demographics (*e.g.*, sex, age, race, and ethnicity), comorbidities (*e.g.*, hypertension, diabetes, chronic kidney disease, atrial fibrillation, stroke, dyslipidemia, nicotine dependence, alcohol use disorders, and overweight/obesity), medications (*e.g.*, lipid-lowering agents, loop diuretics, calcium channel blockers, digoxin, anti-arrhythmic therapies, metformin, insulin, anticoagulation, and anti-platelet therapies), and laboratory data (*e.g.*, left ventricular ejection fraction, NT-proBNP, HbA1c, body mass index, and serum creatinine).

#### 2.2.1 Inclusion Criteria

The inclusion criteria were as follows:

◻ Adults aged 18 years or older.
◻ Diagnosis of heart failure with reduced ejection fraction (HFrEF).
◻ Patients treated with an SGLT2 inhibitor.
◻ Patients receiving guideline-directed medical therapy (GDMT), defined in the manuscript as:
  o    ACE inhibitor / ARB / sacubitril-valsartan
  o    mineralocorticoid receptor antagonist (spironolactone or eplerenone)
  o    beta-blocker (metoprolol or carvedilol)
◻ Availability of data recorded between January 1, 2012, and December 31, 2022.
◻ Patients were stratified by sex (male vs female) for comparison.

#### 2.2.2 Exclusion Criteria

◻ Patients were excluded if they:

◻ Were younger than 18;
◻ Did not have HFrEF;
◻ Were not on SGLT2 inhibitors;
◻ Were not on the specified GDMT regimen; or
◻ Lacked the coding definitions used to identify the study population/outcomes

### 2.3 Study Endpoints

Outcomes were evaluated over a one-year follow-up time. Primary outcomes included all-cause mortality and acute HF events. Secondary endpoints consisted of all-cause hospitalizations, atrial fibrillation or flutter, cardiac arrest, heart transplant, left ventricular assist device use, urinary tract infections, and acute renal failure or need for renal replacement therapy. Electronic health record codes to identify the patients of interest and outcomes are described in the supplementary information.

### 2.4 Statistical Analysis

Patients were stratified based on sex, with all using SGLT2is and optimal GDMT. Continuous variables were summarized as mean ± standard deviation (SD) and compared using independent-sample t-tests, while categorical variables were reported as frequencies (n, %) and analyzed using chi-square (χ^2^) tests. PSM was conducted using a 1:1 nearest-neighbor algorithm with a caliper of 0.1 pooled SDs, achieving a standardized mean difference <0.1 for matched variables. Adjusted odds ratios (aORs) with 95% confidence intervals (CIs) were calculated for both primary and secondary outcomes. We plotted Kaplan-Meier survival curves and performed log-rank tests for survival analyses. Cox proportional hazard models were used to estimate hazard ratios (HR) to compare the sex-specific differences in event rates. Statistical significance was defined as a two-sided *p*-value <0.05. Data processing and analyses were conducted using the TriNetX platform and R software.

## 3 RESULTS

Prior to PSM, the male cohort included 37,512 patients, while the female cohort comprised 18,556 patients. After matching, a total of 17,408 patients were included in each cohort. The mean follow-up time was 319.9 days [standard deviation (SD) 100.^6^] for males and 322.7 days (SD: 97.4) for females. Prior to PSM, significant differences were observed in the baseline characteristics of both cohorts (Table **1**). Male patients were more likely to be White and have atrial fibrillation and a history of nicotine dependence or alcohol-related disorders. Female patients were more likely to belong to the Black race and have overweight/obesity and chronic kidney disease. After matching, baseline characteristics were well-balanced between both cohorts (Table **2**).

### 3.1 Primary Outcomes

There were no significant differences in all-cause mortality between male (n=1,311, 7.5%) and female (n=1,307, 7.5%) cohorts (aOR: 1.003, 95% CI: 0.926–1.087) with a survival probability at the end of the study period of 91.81% for males and 91.92% for females (Table **3**). Male patients (n=6,826, 39.2%) demonstrated lower odds of acute HF events (aOR: 0.949, 95% CI: 0.909–0.991) compared to female patients (n=7,044, 40.5%). By the end of the study period, the acute HF-free survival probabilities were 58.77% for males and 57.68% for females (HR: 0.970, 95% CI: 0.938–1.003) (Fig. **1**).

### 3.2 Secondary Outcomes

Males (n=8,397, 48.2%) had lower odds of all-cause hospitalization compared to females (n=8, 698, 50.0%) (aOR: 0.933, 95% CI: 0.895–0.973), with hospitalization-free survival rates of 49.82% for males and 48.12% for females (HR: 0.961, 95% CI: 0.932–0.990) (Fig. **2**). Males (n=6,079, 34.9%) were more likely to develop atrial fibrillation/flutter than females (n=5, 659, 32.5%) (aOR: 1.114, 95% CI: 1.066–1.165), with atrial fibrillation/flutter-free survival of 63.05% for males *versus* 65.89% for females (HR: 1.097, 95% CI: 1.058–1.137). No significant sex differences were observed in cardiac arrest outcomes (aOR: 0.903, 95% CI: 0.785–1.039).

Male patients had lower odds of renal failure (n=3,741, 21.5%) compared to females (n=4,009, 23.0%) (aOR: 0.915, 95% CI: 0.870–0.962), with renal failure-free survival rates, which favored male patients (76.79%) over female patients (75.35%) (HR: 0.931, 95% CI: 0.891–0.974) (Fig. **3**). Urinary tract infections were more common in females (n=1,851, 10.6%) than males (n=747, 4.3%) (aOR: 0.377, 95% CI: 0.345-0.411), with urinary tract infection-free survival of 95.24% for males and 88.39% for females (HR: 0.393, 95% CI: 0.361–0.428).

Odds of heart transplantation were similar between sexes (males: n=302, 1.7%; females: n=260, 1.5%) (aOR: 1.164, 95% CI: 0.985–1.376), but males had higher odds of LVAD implantation (n=106, 0.6%) compared to females (n=75, 0.4%) (aOR: 1.416, 95% CI: 1.053–1.905).

## 4 DISCUSSION

HFrEF is a debilitating condition associated with significant mortality and morbidity [15]. SGLT2is have emerged as a cornerstone in the management of HFrEF, now recognized as one of the four foundational pillars of care [2-5]. While evidence supports the role of SGLT2is in reducing cardiovascular mortality, adverse cardiovascular events, and hospitalizations [16], there remains a notable gap in understanding potential sex-based differences in cardiovascular outcomes among patients with HFrEF treated with these therapies. Addressing this gap, our study has represented the first real-world cohort of this size to investigate sex-specific impacts of SGLT2is on HFrEF-related outcomes. Our findings have provided valuable population-level data highlighting differences in heart failure-related outcomes between male and female patients treated with SGLT2is.

A recent meta-analysis of three major randomized controlled trials (EMPA-REG, CANVAS, and DECLARE-TIMI) reported a significant reduction in MACE in males treated with SGLT2is (HR: 0.88; 95% CI: 0.82–0.93; *p* = 0.006), but not in females [17]. However, this meta-analysis primarily focused on patients with diabetes rather than those with HFrEF. In contrast, a secondary analysis of the DAPA-HF and DELIVER trials examined sex-based differences in heart failure outcomes among patients treated with dapagliflozin across a spectrum of left ventricular ejection fractions [18]. This study found that female patients were less likely than male patients to experience cardiovascular-related death, all-cause death, or heart failure hospitalizations. In contrast to these findings, our study observed a different trend; male patients treated with SGLT2is had lower rates of acute heart failure events and all-cause hospitalizations compared to female patients.

Several factors may explain these differences. First, while all cohorts in our study received optimal GDMT, including SGLT2 inhibitors, we were unable to ascertain dosing parameters. Previous studies have suggested that pharmacological inertia and clinician hesitancy to up-titrate GDMT in female patients may adversely impact their heart failure outcomes [19-21]. Second, the timing of GDMT initiation may also be crucial. Earlier initiation is associated with better outcomes, whereas delayed initiation or up-titration, possibly due to clinician hesitancy, could disproportionately affect female patients with HFrEF [14, 22]. Third, consistent with previous research, female patients often present with higher rates of age-related comorbidities, a predisposition to coronary microvascular dysfunction, and endothelial inflammation [23, 24]. Additionally, female patients frequently report worse baseline functional and self-reported health status compared to male patients. Despite careful cohort matching to balance these variables, inherent differences in the progression of HFrEF among female patients likely influenced our findings. Although these factors have provided plausible explanations for the observed sex-based inconsistencies in outcomes, they have also emphasized the multifactorial nature of these disparities. Further investigation is needed to better understand and address the underlying mechanisms for these disparities.

Our analysis found similar heart transplant rates between male and female patients treated with SGLT2is. However, LVAD use was higher in males. This contrasts with prior US studies that showed disparities in heart transplantation, where females were less likely to receive transplants and had higher rates of being removed from transplant lists due to worsening health [25], while being similar to prior LVAD studies showing male predominance [26]. Several factors may contribute to these disparities, including lower referral rates of females to advanced heart failure specialists, implicit bias among healthcare providers influencing eligibility decisions, and higher rates of allosensitization in females, particularly those with prior pregnancies, which may result in longer waitlist times and increased waitlist mortality [25].

Female participants remain underrepresented in clinical trials, despite the comparable prevalence of heart failure among males and females [27]. A review of 740 cardiovascular studies reported that females comprised only 38.2% of participants, resulting in HF treatment guidelines being predominantly informed by data derived from male patients [27]. Females with HFrEF are often more symptomatic than men, but they are less likely to receive GDMT [28]. This underrepresentation in clinical trials has significantly hindered our understanding of sex-specific responses to HF treatments, highlighting the importance and novelty of our study's objectives.

## CONCLUSION

This study found potential sex-specific differences in cardiovascular outcomes for HFrEF patients treated with SGLT2 inhibitors. While mortality rates were similar between sexes, male patients demonstrated lower odds of acute heart failure events, all-cause hospitalizations, and renal failure compared to female patients. Importantly, the higher rates of LVAD implantation observed in males raise additional questions about potential differences in disease progression or referral pathways for advanced therapies. Given the increasing utilization of SGLT2is across the spectrum of heart failure, these data highlight the need for sex-disaggregated analyses in both clinical trials and real-world studies. These findings emphasize the need for further research to highlight the mechanisms underlying these disparities and inform more equitable and effective treatment strategies.

## STUDY LIMITATIONS

Like other studies using large healthcare databases, our retrospective cohort study has involved several limitations. The TriNetX platform is susceptible to errors, including miscoding and incomplete reporting of medications, diagnoses, and procedures. The use of electronic health records inherently limits the capture of certain clinical details, such as patient-reported outcomes, physician evaluations, and treatments received outside hospital settings. Consequently, our ability to assess the impact of SGLT2 inhibitors on quality-of-life outcomes was limited. Additionally, we were unable to obtain specific dosing information, adherence, or up-titration measures for the pharmacological regimens used, as well as the severity of heart failure at the initiation of GDMT. Although we utilized propensity score matching to address covariate imbalances, residual confounding from unmeasured clinical factors, such as comorbidity burden, could not be entirely excluded.

## Figures and Tables

**Fig. (1):**
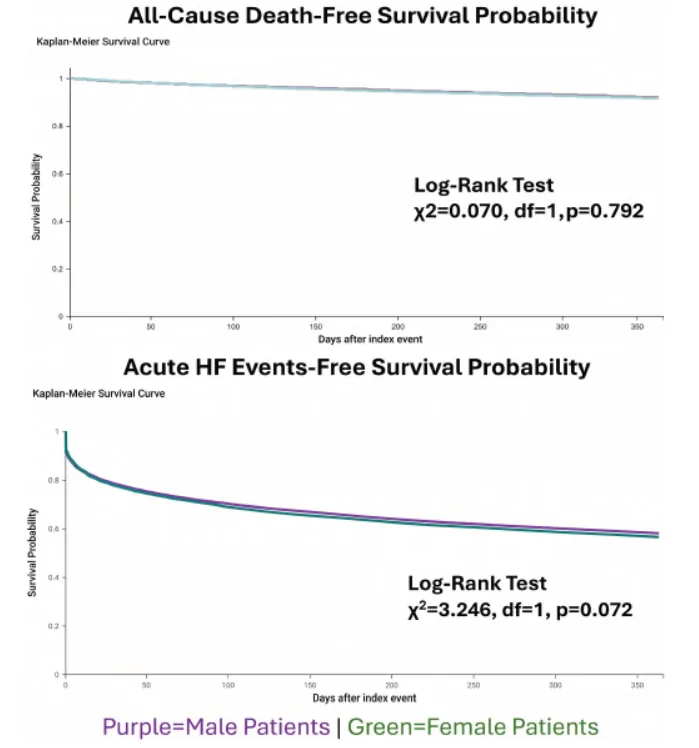
Event-free survival probabilities. The figure depicts the all-cause death and acute heart failure event-free survival probabilities by the end of the one-year study duration. **Abbreviation:** HF = Heart failure.

**Fig. (2):**
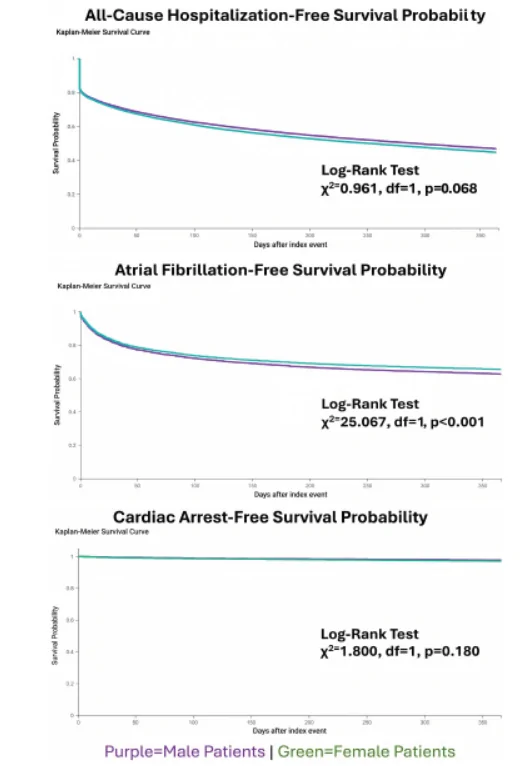
Event-free survival probabilities. The figure depicts all-cause hospitalization, atrial fibrillation/flutter, and cardiac arrest-free survival probabilities by the end of the one-year study duration.

**Fig. (3):**
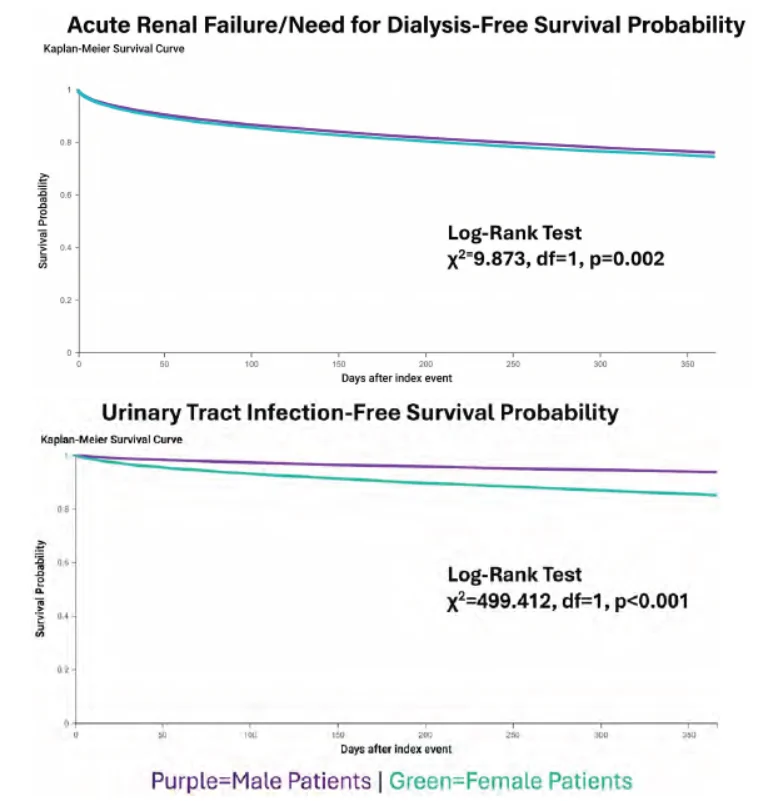
Event-free survival probabilities. The figure depicts the acute renal failure/RRT requirement and UTI-free survival probabilities by the end of the one-year study duration. **Abbreviations:** RRT = Renal replacement therapy, UTI = Urinary tract infection.

**Table 1:** Pre-matching baseline demographics.

**Variable**	**Patient Count (%) – Male **	**Patient Count (%) - Female**	***p*-value**	**Std. Diff.**
Age (mean ± SD)	65.8 +/- 12.8	66.1 +/- 13.8	0.003	0.026
White	22,169 (59.1%)	9,268 (49.9%)	<0.001	0.185
Black or African American	8,235 (22.0%)	5,961 (32.1%)	<0.001	0.231
Hispanic or Latino	2,494 (6.6%)	1,167 (6.3%)	0.105	0.015
Atrial fibrillation and flutter	17,070 (45.5%)	6,659 (35.9%)	<0.001	0.197
Hypertensive diseases	32,226 (85.9%)	16,005 (86.3%)	0.269	0.01
Ischemic heart diseases	28,692 (76.5%)	12,673 (68.3%)	<0.001	0.184
Cerebral infarction	4,432 (11.8%)	2,226 (12.0%)	0.532	0.006
Diabetes mellitus	22,204 (59.2%)	11,116 (59.9%)	0.105	0.015
Overweight and obesity	16,547 (44.1%)	9,743 (52.5%)	<0.001	0.169
Disorders of lipoprotein metabolism	28,005 (74.7%)	13,579 (73.2%)	<0.001	0.034
Chronic kidney disease	13,936 (37.2%)	6,327 (34.1%)	<0.001	0.064
Personal history of nicotine dependence	13,229 (35.3%)	5,385 (29.0%)	<0.001	0.134
Sleep apnea	13,318 (35.5%)	6,001 (32.3%)	<0.001	0.067
Alcohol-related disorders	4,452 (11.9%)	877 (4.7%)	<0.001	0.261
Loop diuretics	32,005 (85.3%)	16,273 (87.7%)	<0.001	0.07
Angiotensin receptor/neprilysin inhibitor	17,968 (47.9%)	8,183 (44.1%)	<0.001	0.076
Antilipemic agents	31,229 (83.3%)	14,610 (78.7%)	<0.001	0.115
Calcium channel blockers	18,801 (50.1%)	9,783 (52.7%)	<0.001	0.052
Digoxin	6,476 (17.3%)	2,994 (16.1%)	0.001	0.03
Anti-arrhythmics	28,252 (75.3%)	14,195 (76.5%)	0.002	0.028
Metformin	14,080 (37.5%)	7,169 (38.6%)	0.012	0.023
Insulin	20,802 (55.5%)	10,290 (55.5%)	0.999	<0.001
Anti-platelet medications	30,034 (80.1%)	14,140 (76.2%)	<0.001	0.094
Anticoagulation	32,097 (85.6%)	15,525 (83.7%)	<0.001	0.053
Hemoglobin A1c (mean ± SD)	7.1 +/- 1.9	7.1 +/- 2.0	0.078	0.018
BMI (mean ± SD)	31.7 +/- 7.7	33.2 +/- 9.4	<0.001	0.180
Creatinine (mean ± SD)	1.2 +/- 1.2	1.1 +/- 1.9	<0.001	0.100
LVEF (mean ± SD)	35.1 +/- 14.5	39.4 +/- 15.5	<0.001	0.286
NT-proBNP (mean ± SD)	3516.4 +/- 5774.8	3912.8 +/- 6966.4	<0.001	0.062

**Note: **The table depicts both male and female cohorts with HFrEF that were on optimal GDMT, including SGLT2is use, prior to propensity score matching.

**Table 2:** Propensity score matching baseline demographics.

**Variable**	**Patient Count (%) - Male**	**Patient Count (%) - Female**	***p*-value**	**Std. Diff.**
Age (mean ± SD)	66.0 ± 12.9	66.1 ± 13.8	0.582	0.006
White	9,076 (52.1%)	9,074 (52.1%)	0.983	<0.001
Black or African American	5,196 (29.8%)	5,134 (29.5%)	0.467	0.008
Hispanic or Latino	1,093 (6.3%)	1,121 (6.4%)	0.539	0.007
Atrial fibrillation and flutter	6,494 (37.3%)	6,491 (37.3%)	0.973	<0.001
Hypertensive diseases	15,013 (86.2%)	14,939 (85.8%)	0.253	0.012
Ischemic heart diseases	12,859 (73.9%)	11,959 (68.7%)	<0.001	0.114
Cerebral infarction	2,062 (11.8%)	2,065 (11.9%)	0.96	0.001
Diabetes mellitus	10,378 (59.6%)	10,386 (59.7%)	0.93	0.001
Overweight and obesity	8,789 (50.5%)	8,791 (50.5%)	0.983	<0.001
Disorders of lipoprotein metabolism	12,778 (73.4%)	12,751 (73.2%)	0.744	0.004
Chronic kidney disease	6,005 (34.5%)	5,982 (34.4%)	0.795	0.003
Personal history of nicotine dependence	5,194 (29.8%)	5,182 (29.8%)	0.888	0.002
Sleep apnea	5,666 (32.5%)	5,712 (32.8%)	0.599	0.006
Alcohol-related disorders	919 (5.3%)	876 (5.0%)	0.297	0.011
Loop diuretics	15,205 (87.3%)	15,176 (87.2%)	0.641	0.005
Angiotensin receptor/neprilysin inhibitor	7,781 (44.7%)	7,816 (44.9%)	0.683	0.004
Antilipemic agents	13,830 (79.4%)	13,857 (79.6%)	0.72	0.004
Calcium channel blockers	9,046 (52.0%)	9,048 (52.0%)	0.983	<0.001
Digoxin	2,880 (16.5%)	2,854 (16.4%)	0.707	0.004
Anti-arrhythmics	13,185 (75.7%)	13,187 (75.8%)	0.98	<0.001
Metformin	6,645 (38.2%)	6,675 (38.3%)	0.741	0.004
Insulin	9,598 (55.1%)	9,626 (55.3%)	0.763	0.003
Anti-platelet medications	13,342 (76.6%)	13,363 (76.8%)	0.79	0.003
Anticoagulation	14,578 (83.7%)	14,568 (83.7%)	0.885	0.002
Hemoglobin A1c (mean ± SD)	7.2 ± 2.0	7.1 ± 2.0	0.14	0.019
BMI (mean ± SD)	32.2 ± 8.0	33.0 ± 9.4	<0.001	0.092
Creatinine (mean ± SD)	1.2 ± 0.5	1.1 ± 1.9	<0.001	0.079
LVEF (mean ± SD)	37.6 ± 15.1	38.8 ± 15.4	<0.001	0.082
NT-proBNP (mean ± SD)	3,373.8 ± 5,502.1	3,998.8 ± 7,070.2	<0.001	0.099

**Note:** The table depicts both male and female cohorts with HFrEF that were on optimal GDMT, including SGLT2is use, post-propensity score matching.

**Table 3:** Outcomes.

**Outcome**	**Patient Count (%) - Male**	**Patient Count (%) - Female**	**Adjusted Odds Ratio (95% CI)**	**Hazard Ratio (95% CI)**
**Primary Outcomes**				
Acute HF	6,826 (39.2%)	7,044 (40.5%)	0.949 (0.909-0.991)	0.970 (0.938-1.003)
All-cause mortality	1,311 (7.5%)	1,307 (7.5%)	1.003 (0.926-1.087)	1.010 (0.936-1.091)
**Secondary Outcomes**				
All-cause hospitalization	8,397 (48.2%)	8,698 (50.0%)	0.933 (0.895-0.973)	0.961 (0.932-0.990)
Cardiac arrest	383 (2.2%)	423 (14.2%)	0.903 (0.785-1.039)	0.910 (0.792-1.045)
Heart transplant	302 (1.7%)	260 (1.5%)	1.164 (0.985-1.376)	1.171 (0.992-1.382)
LVAD	106 (0.6%)	75 (0.4%)	1.416 (1.053-1.905)	1.425 (1.060-1.916)
ESRD, RRT, renal failure	3,741 (21.5%)	4,009 (23.0%)	0.915 (0.870-0.962)	0.931 (0.891-0.974)
Atrial fibrillation/flutter	6,079 (34.9%)	5,659 (32.5%)	1.114 (1.066-1.165)	1.097 (1.058-1.137)
UTI	747 (4.3%)	1,851 (10.6%)	0.377 (0.345-0.411)	0.393 (0.361-0.428)

**Note: **The table depicts the primary and secondary outcomes in both male and female cohorts with HFrEF that were on optimal GDMT, including SGLT2is use.

## Data Availability

All data in an aggregated format is publicly available through an institutional agreement with the TriNetX database.

## LIST OF ABBREVIATIONS

GDMT: Guideline-directed Medical Therapy
HfrEF: Heart Failure with Reduced Ejection Fraction
LVAD: Left Ventricular Assist Device
MACE: Major Adverse Cardiovascular Event
SGLT2is: Sodium-glucose Transport Protein 2 Inhibitors
T2DM: Type 2 Diabetes Mellitus

## STANDARDS OF REPORTING

STROBE guidelines were followed.

## DECLARATION

Graphical abstract was created using Biorender.com.
