# Supplementary material for: SGLT2 Inhibitors in Heart Failure with Reduced Ejection Fraction: A Retrospective Cohort Analysis of Sex-Specific Cardiovascular Outcomes
Source: Curr Cardiol Rev. 2025 Oct 2;22(4):E1573403X394902. doi: 10.2174/011573403X394902250911111958 (PMC13519579; doi:10.2174/011573403X394902250911111958)
Supplement: Supplementary file 1 [file CCR-22-4-E1573403X394902_SD1.pdf]

Supplementary Material

SGLT2 Inhibitors in Heart Failure with Reduced Ejection Fraction: A Retrospective Cohort Analysis of Sex-Specific Cardiovascular Outcomes

Ramzi Ibrahim[1,*], Rahmeh Al-Asmar[2], Hashim AlHammouri[2], Mahmoud Abdelnabi[1], Beani Forst[1], Hoang Nhat Pham[3,4], Patrick Sarkis[1], Steven J. Lester[1], Chadi Ayoub[1], Kwan Lee[1], Julie Rosenthal[1] and Reza Arsanjani[1]

[1]Department of Cardiovascular Medicine, Mayo Clinic, Phoenix, Arizona; [2]Faculty of Medicine, University of Jordan, Amman, Jordan; [3]Department of Medicine, University of Arizona, Tucson, Arizona; [4]Department of Cardiovascular Medicine, Mayo Clinic, Rochester, Minnesota

Query Criteria for the Male Cohort

**Ungrouped terms**					
	must have		demographics	Age	Age (at least 18 years (most recent occurrence))
		and	demographics	UMLS:HL7V3.0:Gender:M	Male
		and	demographics	UMLS:HL7V3.0:Gender:M	Male
		and any of	medication	NLM:VA:CV800	ACE INHIBITORS
			medication	NLM:VA:CV805	ANGIOTENSIN II INHIBITOR
			medication	NLM:RXNORM:1656328	sacubitril
		and any of	medication	NLM:RXNORM:20352	carvedilol
			medication	NLM:RXNORM:6918	metoprolol
		and any of	medication	NLM:RXNORM:298869	eplerenone
			medication	NLM:RXNORM:9997	spironolactone
			medication	NLM:VA:CV805	ANGIOTENSIN II INHIBITOR
			medication	NLM:RXNORM:1656328	sacubitril
		and any of	medication	NLM:RXNORM:20352	carvedilol
			medication	NLM:RXNORM:6918	metoprolol
		and any of	medication	NLM:RXNORM:298869	eplerenone
			medication	NLM:RXNORM:9997	spironolactone
**Group 1**					
	Group 1A				
	must have		diagnosis	UMLS:ICD10CM:I50.2	Systolic (congestive) heart failure
		and any of	medication	NLM:RXNORM:1488564	dapagliflozin
			medication	NLM:RXNORM:1545653	empagliflozin
			medication	NLM:RXNORM:1373458	canagliflozin
	must have		diagnosis	UMLS:ICD10CM:I50.2	Systolic (congestive) heart failure
		and any of	medication	NLM:RXNORM:1488564	dapagliflozin

			medication	NLM:RXNORM:1545653	empagliflozin
			medication	NLM:RXNORM:1373458	canagliflozin
	date constraint		The terms in this group occurred between Jan 1, 2012 and Dec 31, 2022		

Query Criteria for the Female Cohort

Ungrouped terms					
	must have		demographics	Age	Age (at least 18 years (most recent occurrence))
		and	demographics	UMLS:HL7V3.0:Gender:F	Female
		and	demographics	UMLS:HL7V3.0:Gender:F	Female
		and any of	medication	NLM:VA:CV800	ACE INHIBITORS
			medication	NLM:VA:CV805	ANGIOTENSIN II INHIBITOR
			medication	NLM:RXNORM:1656328	sacubitril
		and any of	medication	NLM:RXNORM:20352	carvedilol
			medication	NLM:RXNORM:6918	metoprolol
		and any of	medication	NLM:RXNORM:298869	eplerenone
			medication	NLM:RXNORM:9997	spironolactone
			medication	NLM:VA:CV805	ANGIOTENSIN II INHIBITOR
			medication	NLM:RXNORM:1656328	sacubitril
		and any of	medication	NLM:RXNORM:20352	carvedilol
			medication	NLM:RXNORM:6918	metoprolol
		and any of	medication	NLM:RXNORM:298869	eplerenone
			medication	NLM:RXNORM:9997	spironolactone
**Group 1**					
	Group 1A				
	must have		diagnosis	UMLS:ICD10CM:I50.2	Systolic (congestive) heart failure
		and any of	medication	NLM:RXNORM:1488564	dapagliflozin
			medication	NLM:RXNORM:1545653	empagliflozin
			medication	NLM:RXNORM:1373458	canagliflozin
	must have		diagnosis	UMLS:ICD10CM:I50.2	Systolic (congestive) heart failure
		and any of	medication	NLM:RXNORM:1488564	dapagliflozin
			medication	NLM:RXNORM:1545653	empagliflozin
			medication	NLM:RXNORM:1373458	canagliflozin
	date constraint		The terms in this group occurred between Jan 1, 2012 and Dec 31, 2022		

Outcome Definitions

**all cause death**			
**Outcome definition**			
	Demographics	Deceased	Deceased
**Settings for the performed analyses**			
	Risk analysis		including patients with outcome prior to the time window
	Kaplan - Meier survival analysis		including patients with outcome prior to the time window
	Number of instances analysis		including patients with outcome prior to the time window<br>excluding patients with zero outcomes<br>counts are grouped by date
**all cause hospitalization**			
**Outcome definition**			
	Visit	UMLS:HL7V3.0:VisitType:IMP	Visit: Inpatient Encounter
	Visit	UMLS:HL7V3.0:VisitType:NONAC	Visit: Inpatient Non-acute
	Visit	UMLS:HL7V3.0:VisitType:ACUTE	Visit: Inpatient Acute
	Procedure	UMLS:CPT:1013659	Hospital Inpatient and Observation Care Services
	Procedure	UMLS:CPT:1013729	Critical Care Services
	Procedure	UMLS:CPT:1013699	Inpatient or Observation Consultations
	Visit	UMLS:HL7V3.0:VisitType:OBSENC	Visit: Observation Encounter
	Visit	UMLS:HL7V3.0:VisitType:SS	Visit: Short Stay
**Settings for the performed analyses**			
	Risk analysis		including patients with outcome prior to the time window
	Number of instances analysis		including patients with outcome prior to the time window<br>excluding patients with zero outcomes<br>counts are grouped by date
	Kaplan - Meier survival analysis		including patients with outcome prior to the time window
**acute HF**			
**Outcome definition**			
	Diagnosis	UMLS:ICD10CM:I50.21	Acute systolic (congestive) heart failure
	Diagnosis	UMLS:ICD10CM:I50.23	Acute on chronic systolic (congestive) heart failure
	Diagnosis	UMLS:ICD10CM:I50.33	Acute on chronic diastolic (congestive) heart failure
	Diagnosis	UMLS:ICD10CM:I50.41	Acute combined systolic (congestive) and diastolic (congestive) heart failure
	Diagnosis	UMLS:ICD10CM:I50.43	Acute on chronic combined systolic (congestive) and diastolic (congestive) heart failure
	Medication	NLM:VA:CV702	LOOP DIURETICS (Route: Injectable Product)
	Diagnosis	UMLS:ICD10CM:I50.31	Acute diastolic (congestive) heart failure
**Settings for the performed analyses**			
	Kaplan - Meier survival analysis		including patients with outcome prior to the time window
	Risk analysis		including patients with outcome prior to the time window

	Number of instances analysis		including patients with outcome prior to the time window<br>excluding patients with zero outcomes<br>counts are grouped by date
**cardiac arrest**			
	**Outcome definition**		
	Diagnosis	UMLS:ICD10CM:I46	Cardiac arrest
	**Settings for the performed analyses**		
	Risk analysis		including patients with outcome prior to the time window
	Number of instances analysis		including patients with outcome prior to the time window<br>excluding patients with zero outcomes<br>counts are grouped by date
	Kaplan - Meier survival analysis		including patients with outcome prior to the time window
**heart transplant**			
	**Outcome definition**		
	Diagnosis	UMLS:ICD10CM:Z94.1	Heart transplant status
	Procedure	UMLS:CPT:33945	Heart transplant, with or without recipient cardiectomy
	**Settings for the performed analyses**		
	Risk analysis		including patients with outcome prior to the time window
	Kaplan - Meier survival analysis		including patients with outcome prior to the time window
	Number of instances analysis		including patients with outcome prior to the time window<br>excluding patients with zero outcomes<br>counts are grouped by date
**LVAD**			
	**Outcome definition**		
	Procedure	UMLS:SNOMED:232967006	LVAD - Implantation of left ventricular assist device
	**Settings for the performed analyses**		
	Kaplan - Meier survival analysis		including patients with outcome prior to the time window
	Number of instances analysis		including patients with outcome prior to the time window<br>excluding patients with zero outcomes<br>counts are grouped by date
	Risk analysis		including patients with outcome prior to the time window
**ESRD, RRT, RENAL FAILURE**			
	**Outcome definition**		
	Diagnosis	UMLS:ICD10CM:N17	Acute kidney failure
	Diagnosis	UMLS:ICD10CM:N18.6	End stage renal disease
	Procedure	UMLS:CPT:1012740	Dialysis Services and Procedures
	Procedure	UMLS:CPT:90945	Dialysis procedure other than hemodialysis (eg, peritoneal dialysis, hemofiltration, or other continuous renal replacement therapies), with single evaluation by a physician or other qualified health care professional
	Procedure	UMLS:CPT:90947	Dialysis procedure other than hemodialysis (eg, peritoneal dialysis, hemofiltration, or other continuous renal replacement therapies) requiring repeated evaluations by a physician or other qualified health care professional, with or without substantial revision of dialysis prescription
	Procedure	UMLS:CPT:1012752	Hemodialysis Procedures

	Procedure	UMLS:CPT:90935	Hemodialysis procedure with single evaluation by a physician or other qualified health care professional
	Procedure	UMLS:CPT:1006747	Hemodialysis Access, Intervascular Cannulation for Extracorporeal Circulation, or Shunt Insertion Procedures on Arteries and Veins
	Procedure	UMLS:ICD9CM:39.95	Hemodialysis
	Procedure	UMLS:CPT:90937	Hemodialysis procedure requiring repeated evaluation(s) with or without substantial revision of dialysis prescription
	Procedure	UMLS:SNOMED:302497006	Hemodialysis
	Procedure	UMLS:CPT:90940	Hemodialysis access flow study to determine blood flow in grafts and arteriovenous fistulae by an indicator method
	Procedure	UMLS:SNOMED:233586004	Hemodiafiltration
	Procedure	UMLS:HCPCS:C1750	Catheter, hemodialysis/peritoneal, long-term
	Procedure	UMLS:HCPCS:C1752	Catheter, hemodialysis/peritoneal, short-term
	Procedure	UMLS:SNOMED:233578004	Continuous hemodialysis
	Procedure	UMLS:CPT:99512	Home visit for hemodialysis
	**Settings for the performed analyses**		
	Risk analysis		including patients with outcome prior to the time window
	Kaplan - Meier survival analysis		including patients with outcome prior to the time window
	Number of instances analysis		including patients with outcome prior to the time window<br>excluding patients with zero outcomes<br>counts are grouped by date
**atrial fibrillation.flutter**			
	**Outcome definition**		
	Diagnosis	UMLS:ICD10CM:I48	Atrial fibrillation and flutter
	**Settings for the performed analyses**		
	Risk analysis		including patients with outcome prior to the time window
	Kaplan - Meier survival analysis		including patients with outcome prior to the time window
	Number of instances analysis		including patients with outcome prior to the time window<br>excluding patients with zero outcomes<br>counts are grouped by date
**UTI**			
	**Outcome definition**		
	Diagnosis	UMLS:ICD10CM:N39.0	Urinary tract infection, site not specified
	**Settings for the performed analyses**		
	Kaplan - Meier survival analysis		including patients with outcome prior to the time window
	Number of instances analysis		including patients with outcome prior to the time window<br>excluding patients with zero outcomes<br>counts are grouped by date
	Risk analysis		including patients with outcome prior to the time window
	Kaplan - Meier survival analysis		including patients with outcome prior to the time window
	Number of instances analysis		including patients with outcome prior to the time window<br>excluding patients with zero outcomes<br>counts are grouped by date
